# Recent advances in mast cell activation and regulation

**DOI:** 10.12688/f1000research.22037.1

**Published:** 2020-03-19

**Authors:** Hwan Soo Kim, Yu Kawakami, Kazumi Kasakura, Toshiaki Kawakami

**Affiliations:** 1 Division of Cell Biology, La Jolla Institute for Immunology, La Jolla, California, 92037, USA; 2 Department of Pediatrics, College of Medicine, The Catholic University of Korea, Seoul, South Korea; 3 Department of Dermatlogy, University of California San Diego, School of Medicine, La Jolla, CA, 92093, USA

**Keywords:** Allergy, mast cells, allergen, IgE, FcεRI, MRGPRX2, IL-33, miRNA

## Abstract

Mast cells are innate immune cells that intersect with the adaptive immunity and play a crucial role in the initiation of allergic reactions and the host defense against certain parasites and venoms. When activated in an allergen- and immunoglobulin E (IgE)-dependent manner, these cells secrete a large variety of allergenic mediators that are pre-stored in secretory granules or *de novo*–synthesized. Traditionally, studies have predominantly focused on understanding this mechanism of mast cell activation and regulation. Along this line of study, recent studies have shed light on what structural features are required for allergens and how IgE, particularly anaphylactic IgE, is produced. However, the last few years have seen a flurry of new studies on IgE-independent mast cell activation, particularly via Mrgprb2 (mouse) and MRGPRX2 (human). These studies have greatly advanced our understanding of how mast cells exert non-histaminergic itch, pain, and drug-induced pseudoallergy by interacting with sensory neurons. Recent studies have also characterized mast cell activation and regulation by interleukin-33 (IL-33) and other cytokines and by non-coding RNAs. These newly identified mechanisms for mast cell activation and regulation will further stimulate the allergy/immunology community to develop novel therapeutic strategies for treatment of allergic and non-allergic diseases.

## Introduction

Mast cells (MCs) play a crucial role in allergic reactions and the host defense against certain parasites, bacteria, and venoms. Morphologically, MCs are featured by a large number of secretory granules containing various bioactive molecules, including histamine, serotonin, proteoglycans, and proteases. Upon encounter with multivalent antigen (or allergen), antigen-specific immunoglobulin E (IgE)-bound high-affinity IgE receptors (FcεRI) on the surface of MCs are cross-linked or aggregated. Consequently, activation of the FcεRI signaling system is triggered, leading to the release of granular contents (degranulation) and *de novo* synthesis and secretion of lipid mediators, cytokines, and chemokines. Activation of MCs entails immediate hypersensitivity and late-phase allergic inflammation. With regard to the IgE-mediated MC activation, recent years have seen a deeper understanding of IgE synthesis, structural features of allergens, FcεRI signaling mechanisms, and counter-mechanisms. Non-IgE-dependent MC activation mechanisms have been studied at a slow pace for many years. However, we have witnessed significant progress in this area more recently.

Murine MCs are classified as connective tissue MCs (CTMCs) and mucosal MCs (MMCs) on the basis of their tissue distribution. CTMCs and MMCs are also characterized by the heparin content of their granules: CTMCs contain a large amount of heparin in their granules, whereas MMCs have very little or no heparin. Human MC proteases include tryptases (mMCP-6 and -7 in mouse), chymases (mMCP-1, -2, and -4), an elastase (mMCP-5), and a carboxypeptidase-A3 (CPA3). Human MCs are categorized by expression of MC tryptase (MC _T_) or MC chymase (MC _C_) or both (MC _TC_) ^1^. A recent transcriptional analysis demonstrated that the MC is one of the most transcriptionally variable cell types of the immune system ^2^. Murine MCs that were purified from different tissues shared an “MC-specific” transcriptional signature of at least 100 genes. Also, these MCs showed a tissue-specific regulation of their transcriptomes.

Substantial progress has recently been made in several areas of MC research, such as degranulation machinery, cancer, microbiota, and food allergy. Readers interested in these topics are referred to recent review articles ^3– 8^.

## Allergen, immunoglobulin E, and FcεRI

A comprehensive understanding of the IgE-mediated MC activation requires a better knowledge of allergens, IgE synthesis and structure, and FcεRI structure and signaling pathways. Here, we highlight recent advances in this area, particularly allergens and IgE synthesis. We certainly know three-dimensional structures of many parts of IgE and FcεRI (composed of an IgE-binding α and receptor-stabilizing and signal-amplifying β and activation signal-triggering γ subunits) ^9, 10^ and important principles in signaling, such as tyrosine phosphorylation of β and γ subunits at the immunoreceptor tyrosine-based activation motif (ITAM) by Src family kinases, the essential functions of Syk, Ca ^2+^ flux, several adaptor molecules, mitogen-activated protein kinases (MAPKs), and several transcription factors ^11, 12^.

However, we feel obliged to note that our understanding of FcεRI signaling pathways is still in the early stages in light of an incomplete understanding of degranulation processes and a large number of genes regulated by MC activation.

One of the most important hypotheses on structural features of allergens stemmed from the requirement of cross-linking of cell surface IgE molecules by various allergens for MC activation and IgE synthesis. This line of thinking led Jensen-Jarolim *et al*. to recognize that allergens display repetitive motifs, which they designate allergen-associated molecular patterns ^13, 14^. Indeed, many allergenic molecules occur as dimers or multimers. Some allergens—small proteins, in particular—have just a single immunodominant B-cell epitope and thus do not fulfill the requirement for cross-linking as a single molecular unit. Oligomerization provides the necessary means for efficient IgE cross-linking. Examples where only single dominant epitopes have been found are the allergens Der p 1 from house dust mite (HDM) and Bet v 1 from birch. Also, the occurrence of repetitive epitopes on single native allergen molecules has been shown on high-molecular-weight proteins of wheat and for HDMs and insects, cockroach Bla g 1, latex Hev b 5, and tropomyosin from shrimp.

IgE concentrations in serum are kept to the lowest level among immunoglobulin subtypes by several layers of regulation: in addition to the high rate of turnover, low efficiency of class-switch recombination to IgE, and lower surface expression of membrane IgE than that of IgG1 on germinal center (GC) B cells, IgE ^+^ B cells are predisposed to swiftly exit GCs and differentiate into plasma cells (PCs) and IgE-producing GC B cells die by apoptosis ^15– 17^. Therefore, IgE ^+^ memory B cells are scarce ^18^. Class switching of antigen-specific IgG1 ^+^ cells to become IgE ^+^ cells, via the so-called sequential switching, was proposed as the mechanism involved in the production of affinity-matured IgE antibodies in memory responses ^19^. Using a culture system of induced GC B cells, Haniuda *et al*. showed that the CD19-phosphatidylinositol 3-kinase (PI3K)-Akt-IRF4 axis is the essential pathway for PC differentiation and the BLNK-JNK-p38 axis serves an enhancing role in PC differentiation ^20^. They also showed that BLNK is essential for B-cell apoptosis and that CD19 is rather anti-apoptotic.

Recent studies have shown that T follicular helper (Tfh) cells are the primary T-cell subset responsible for IgE responses. Interleukin-4 (IL-4) is required to generate and sustain IgE production in mice ^21^. In response to allergens, T helper type 2 (Th2) and Tfh cells show unique cytokine responses, tissue localization, and phenotypes. *In vivo*, Tfh cells assist the sustained production of IgE antibody. But conditional deficiency of Bcl6, the master regulator of Tfh, in CD4 ^+^ T cells ^22^ results in a significant decrease in IgE antibody levels and Tfh cell numbers. However, eosinophilic inflammation and type 2 cytokine responses in the airways are not affected. Thus, Tfh-derived IL-4, but not Th2-derived IL-4, is necessary for IgE production ^23^. Gowthaman *et al*. recently discovered a new Tfh subset in mice with T cell–specific Dock8 deficiency ^24^. These mice made allergen-specific anaphylactic IgE with the help of an IL-4– and IL-13–producing Tfh cell population called Tfh13 cells. Tfh13 cells have an unusual cytokine profile (IL-13 ^hi^IL-4 ^hi^IL-5 ^hi^IL-21 ^lo^) and co-express the transcription factors Bcl6 and GATA3. These cells are required for production of high- but not low-affinity IgE and subsequent allergen-induced anaphylaxis. Single-cell RNA sequencing analysis confirmed that Tfh13 cells are distinct from related Th2 or IL-4–expressing Tfh2 cells. Conditional ablation of Tfh13 cells or isolated loss of IL-13 in Tfh cells resulted in impaired anaphylactic IgE responses to allergens. Thus, blocking Tfh cells might represent a therapeutic means to ameliorate anaphylaxis.

We have known effects of monomeric IgE on FcεRI surface levels ^25, 26^ and on survival of MCs ^27, 28^ in the absence of allergen for a long time. A recent study showed that IL-3 but not monomeric IgE regulates FcεRI expression and cell survival in primary human basophils, in contrast to human and murine MCs ^29^.

## Mast cell activation by interleukin-33

IL-33 belongs to the IL-1 family and is expressed by several cell types, including epithelial cells ^30– 33^. IL-33 binds to a specific receptor called T1/ST2 (ST2) that belongs to the Toll-like receptor/IL1R family. ST2 forms heterodimers with the IL-1 receptor accessory protein (IL-1RAcP), namely a transmembrane form (ST2 or ST2L) and a soluble form (sST2). ST2L isoform is expressed on MCs, basophils, Th2 cells, and natural killer cells and coordinates spatially and temporally with IL-33 signaling, which might trigger a key regulatory amplification loop involved in immune homeostasis. IL-33 is considered an alarmin as it is released after necrosis or tissue damage. However, apoptosis leads to the inactivation of IL-33 by cleavage of IL-33 by caspases. In contrast, MC serine proteases cleave the full-length IL-33 (IL-33 _1–270_) and liberate active forms: IL-33 _95–270_, IL-33 _99–270_, and IL-33 _109–270_. These cleaved forms have 10 times greater potency than the full-length protein ^34^. MC chymase also degrades IL-33 that leads to higher bioactivity ^35^. Downstream of ST2, the IL-33–mediated signaling pathway involves MyD88, IRAK1, IRAK4, and TRAF6 as well as activation of MAPKs (ERK1/2, p38, and JNK1/2) and nuclear factor-kappa B (NF-κB) ^36, 37^.

**Figure 1.**
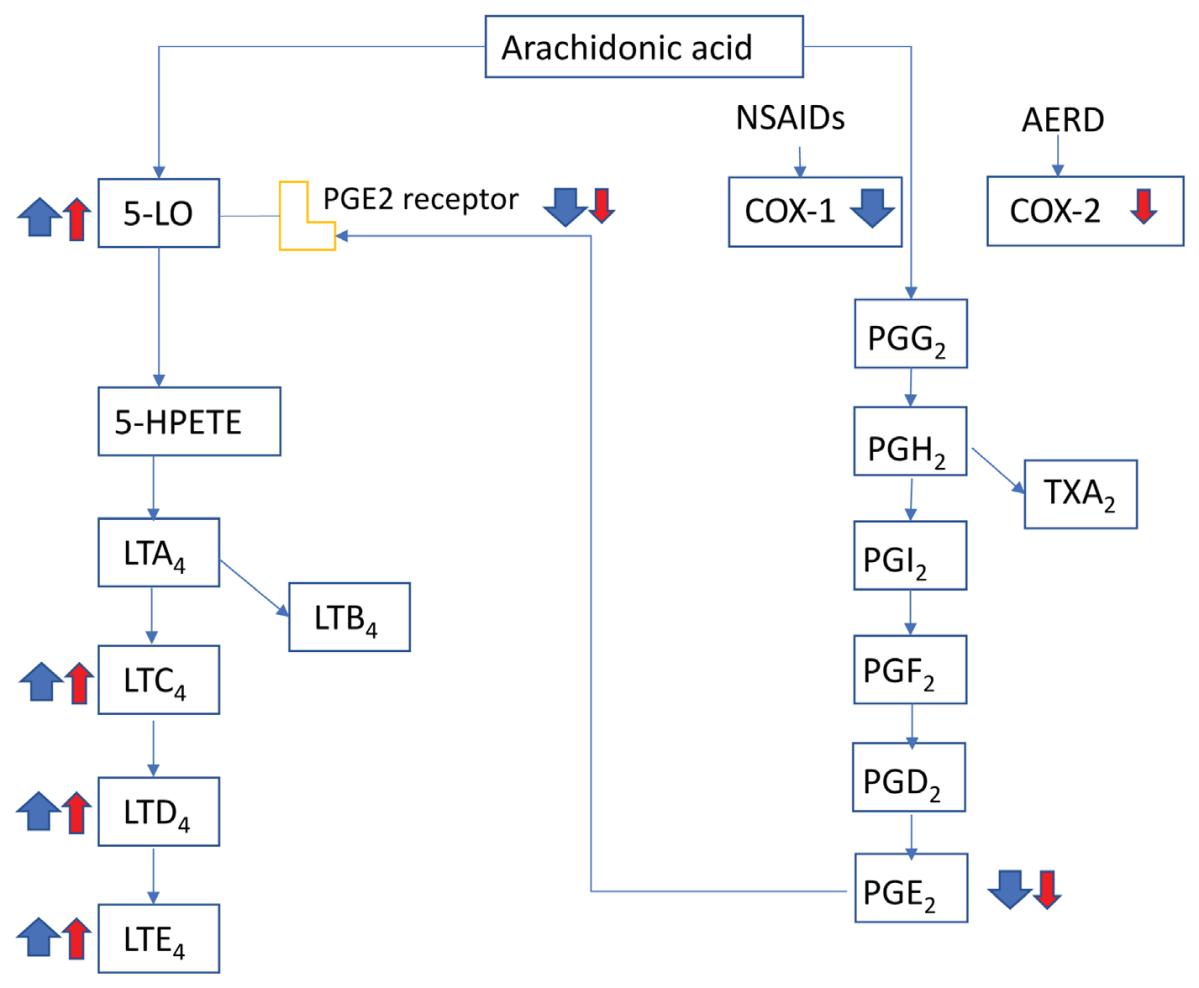
Mechanisms underlying response to cyclooxygenase 1 (COX-1) inhibitors in patients with aspirin-exacerbated respiratory disease (AERD) Patients with AERD show lower levels of COX-2 compared with the healthy population. Non-steroidal anti-inflammatory drugs (NSAIDs) inhibit COX-1 and lower the level of prostaglandin E _2_ (PGE _2_). The loss of PGE _2_ inhibitory control leads to massive release of histamine and generation of cysteinyl leukotrienes by mast cells, an event that is unique to AERD. Red arrows represent abnormal baseline conditions in patients with AERD, and blue arrows indicate changes after COX-1 inhibition. The size of the arrows indicates the magnitude of change. 5-HPETE, 5-hydroperoxyeicosatetraenoic acid; 5-LO, 5-lipoxygenase; LT, leukotriene (A4, C4, D4, and E4); PG, prostaglandin (G2, H2, I2, and F2); TXA2, thromboxane A2.

IL-33 can induce full activation of MCs, including degranulation ^38^ and production of several cytokines and chemokines ^39^, and elicits systemic MC-dependent anaphylaxis ^38^. Several studies have shown that IL-33 plays a significant role in severe asthma ^40^ and refractory nasal polyposis ^41^. Earlier studies have been summarized in excellent reviews ^42– 44^. Here, we touch on newer reports that showed a possible role of IL-33 in various allergic conditions: IL-33–mediated airway constriction was exacerbated through increased secretion of serotonin from MCs ^45^. *Staphylococcus aureus*–derived serine protease-like protein (Spl) D is a potent allergen and induces a Th2-biased inflammatory response in the airways in an IL-33–dependent manner ^46^. Aspirin-exacerbated respiratory disease (AERD) is a severe eosinophilic disorder of the airways and is characterized by overproduction of cysteinyl leukotrienes, activation of airway MCs, and bronchoconstriction in response to non-selective cyclooxygenase inhibitors that deplete prostaglandin E _2_ (PGE _2_) ( Figure 1). A study using clinical samples and mice deficient in PGE _2_ synthase (a model of AERD) found up-regulation of IL-33 in airway epithelium ^47^. Deletion of leukotriene C4 synthase in the AERD model mice eliminates the increased IL-33, lung eosinophilia, and aspirin-induced MC activation and bronchoconstriction. MCs have been shown to play a crucial role in a model of skin inflammation by IL-33–mediated recruitment of leukocytes and resulting inflammation in an MK2/3 (MAPK-activated protein kinases 2 and 3)-dependent manner ^48^. In a murine model of food allergy, IL-33 and MCs promote inflammation in the gastrointestinal tract through IL-4 production by IL-33–stimulated ILC2s, as IL-4 blocks the generation of allergen-specific regulatory T (Treg) cells ^49– 51^. However, on the positive side, IL-33 and MCs play a protective role in intestinal helminth infections by activating ILC2, leading to helminth expulsion ^52^. MCs can ameliorate IL-33–mediated inflammatory effects under certain circumstances. Stimulation of MCs with IL-33 in the absence of IgE cross-linking can induce Treg cell expansion by producing IL-2 and reduce the inflammation in a papain-induced innate-type airway inflammation model ^53^.

## Mast cell activation via Mrgprb2/MRGPRX2

Mas-related G protein–coupled receptor-X2 (MRGPRX2) has been the hottest receptor in MC research over the last few years ^54^. Mrgprb2 is the murine ortholog of MRGPRX2. Under homeostatic conditions, CTMCs in the skin and peritoneum of mice express Mrgprb2, whereas MMCs do not express Mrgprb2 ^55^. Mrgprb2/MRGPRX2 recognizes a wide range of cationic molecules, including substance P (SP), basic secretagogues (for example, compound 48/80), numerous US Food and Drug Administration–approved drugs, and endogenous protein fragments ^55– 57^. Mrgprb2/MRGPRX2-mediated activation of MCs by these ligands results in their rapid degranulation of individual granules and MC-dependent local inflammation, whereas FcεRI-elicited secretion is delayed but progressive and is characterized by granule-to-granule fusions ^58^.

MRGPRX2 has been implicated in allergic and chronic inflammatory diseases. LL-37, the catheliciden peptide and MRGPRX2 agonist ^59, 60^, is up-regulated in rosacea ^61^, and MCs play a key role as the primary source of LL-37 in a murine model of rosacea ^62^. The pathology in asthma ^63^ and urticaria ^64^ correlates with MC-specific expression of MRGPRX2. Mrgprb2 inactive mutant Mrgprb2 ^mut/mut^ mice ^55^ show reduced itch in multiple models of allergic contact dermatitis (ACD), a pruritic inflammatory skin disorder. MC numbers and PAMP1-20 (MRGPRX2 agonist) concentrations are increased in human ACD skin ^65^, which is associated with pathogenic CD8 T-cell responses ^66^. MCs are found in close proximity to peripheral nerve endings ^67– 69^. Atopic dermatitis, another pruritic skin disease, has been studied by using a mouse model sensitized and challenged with HDMs in the presence of staphylococcal enterotoxin B ^70^. Using this model, a recent study shows that HDMs with cysteine protease activity directly activate peptidergic nociceptors on sensory neurons expressing the ion channel TRPV1 and *Tac1* (gene encoding the precursor for SP) ^71^. HDM-activated nociceptors drive the development of allergic skin inflammation by SP/Mrgprb2-mediated activation of MCs ^71^. Another study indicates that activation of the natriuretic polypeptide b (Nppb)-expressing class of sensory neurons elicits scratching responses in mice ^72^. Interestingly, however, Nppb ^+^ neurons express receptors for leukotrienes, serotonin and sphingosine-1-phosphate, and these receptors induce itch by the direct activation of Nppb ^+^ neurons and neurotransmission through the canonical gastrin-releasing peptide-dependent spinal cord itch pathway ^72^. Mrgprb2/MRGPRX2 is also involved in inflammatory mechanical and thermal hyperalgesia ^73^. In this case, SP activates MCs via Mrgprb2/MRGPRX2 to release multiple pro-inflammatory cytokines and chemokines, which facilitate the migration of immune cells. It is noteworthy that SP-mediated activation of MCs does not involve its canonical receptor, neurokinin 1 receptor (NK-1R). However, activation of NK-1R by hemokinin-1 likely contributes to allergic airway inflammation in mice, whereas activation of the human MC line LAD-2 by hemokinin-1 requires MRGPRX2. MRGPRX2 expression is upregulated in lung MCs from patients with lethal asthma ^63^.

Studies of Mrgprb2/MRGPRX2-mediated MC activation have been extended to their new ligands, signal transduction, effects of other MC modulators, and so on. For example, compound 48/80, AG-30/5C (angiogenic defense peptide), and icatibant (bradykinin B2 receptor antagonist) all activate pertussis toxin-sensitive G proteins, but only compound 48/80 activates β-arrestin ^74^. The same study also found resveratrol (polyphenolic compound in peanuts, grapes, red wine, and some berries) as an inhibitor of MRGPRX2. As the FcεRI signaling is initiated by tyrosine phosphorylation with Src, Syk, and Tec family kinases while Mrgprb2 and MRGPRX2 are G protein–coupled receptors, FcεRI- and MRGPRX2-stimulated pathways are completely independent of each other ^75^. Stem cell factor (SCF) and IL-4, which are the two main MC differentiation and growth factors, negatively regulate MRGPRX2 expression in human skin MCs, whereas SCF promotes allergic stimulation via FcεRI ^76^. In contrast, pre-incubation (20 minutes) of human MCs with IL-33 or IL-6 or both does not affect their activation with SP, whereas such priming, particularly that with both IL-33 and IL-6, enhances IgE/allergen-mediated MC activation ^77^. Another study shows that chronic exposure (5 weeks) of human MCs to IL-33 reduces FcεRI expression and responsiveness to its aggregation ^78^. Short-term (30 minutes) pre-incubation with IL-33 enhances MRGPRX2-mediated degranulation by SP or compound 48/80 without changing MRGPRX2 expression, whereas chronic (5 weeks) pre-treatment with IL-33 reduces mRNA and protein expression of MRGPRX2 and its function ^79^. MCs are also required for cardiac fibrosis in multiple animal models. Interestingly, NK-1R expression in MCs is not required in cardiac fibrosis ^80^. It should be tested whether Mrgprb2 is involved in this process.

## MicroRNA and mast cell biology

MicroRNA (miRNA), a small non-coding RNA molecule that is 19 to 25 nucleotides in length, functions in post-transcriptional regulation and RNA silencing of gene expression. miRNAs work by base pairing with complementary sequences inside of mRNA molecules. Because of the broad regulatory mechanisms, miRNAs regulate differentiation, proliferation, survival, apoptosis, stress response, and the effector function as well as the resolution of an immune response ^81, 82^.

Numerous studies have examined the role of miRNAs in MC biology ( Table 1) ^83^. Silencing of Dicer, a key enzyme of miRNA biogenesis, attenuates degranulation, indicating that miRNAs are involved in MC activation. Overexpression of miR-142-3p, which rescues Dicer expression, enhances FcεRI-mediated degranulation in MCs ^84^. IgE/antigen stimulation of bone marrow–derived MCs induces up- or down-regulation of several miRNAs, which affects mRNA expression of some key signaling molecules, including Lyn, Vav3, and Csf2 ^85, 86^. miR-155 plays a critical role in FcεRI-mediated MC responses by modulating components of the PI3Kγ pathway, and miR-155–deficient mice show enhanced anaphylaxis ^87^. Down-regulation of miR-155 in MCs is also involved in suppression of IL-33–induced inflammation by lactic acid ^88^ or of IL-33–induced IL-6 production in MCs ^89^. As a basis of IL-10–mediated MC regulation, IL-10–induced miR-155 expression enhances protease and cytokine production in MCs by suppressing SOCS1, a suppressor of cytokine signaling ^90^. A novel miRNA let7i inhibits MC degranulation by suppressing expression of *Exoc8*, which is an exocytosis-related gene ^91^. MiR-126 accelerates IgE-mediated MC degranulation, which is associated with PI3K/Akt activation and increased Ca ^2+^ influx ^92^. MiR-223 reduces IL-6 secretion in MCs by inhibiting the IGF1R/PI3K signaling pathway ^93^. Expression of miR-210 and miRNA-132/212 cluster is increased by IgE-mediated MC activation ^94^. MiR-21 inhibits MC degranulation by inhibiting the p38 pathway in a murine model of ACD ^95^. MiR-221-222 is up-regulated in MC stimulation and regulates the cell cycle by inhibiting p27 ^Kip1^ expression ^96, 97^. MiR-221-3p, which is markedly increased in asthmatics, up-regulates IL-4 secretion from MCs by targeting phosphatase and tensin homolog (PTEN) as well as activation of p38 and NF-κB ^98^. MiR-302e negatively regulates RelA/p65 expression in MCs and ameliorates allergic inflammation through inhibition of the NF-κB signaling pathway ^99^. MiR-143 and miR-146 reduce MC activation by targeting IL-13Rα1 and TRAF/IRAK, respectively, leading to a reduced allergic response ^100– 103^. miR-20a inhibits expression of tumor necrosis factor (TNF), IL-1β, and interferon gamma (IFN-γ) while promoting IL-10 in HMC-1 human MCs. miR-20a also targets histone deacetylase 4 (HDAC4), which contributes to the epigenetic regulation of IL-10 expression ^104^.

**Table 1.** MicroRNA (miRNA) functions in mast cell activation and proliferation.

miRNA	Trigger	miRNA effect on mast cells	Target mRNA	References
miR-142	FcεRI	Increase degranulation		81
miR-155	FcεRI IL-33 IL-10	Ca ^2+^ influx with degranulation Increase cytokine production Increase cytokine production	PI3K SOCS1	84– 87
Let-7i		Decrease degranulation	Exoc8	88
miR-126	FcεRI	Increases degranulation		89
miR-223	FcεRI	Decrease granulation and interluekin-6 (IL-6) release	IGF1R	90
miR-210	FcεRI			91
miR-132/212	FcεRI		HB-EGF	91
miR-21	Allergic inflammation	Decrease degranulation and IL-12 production	IL-12p35 P38	92
miR-221/222	FcεRI	Regulate proliferation and cell cycle Increases degranulation and cytokine production; reduces migration	p27 ^Kip1^, PTEN	93– 95
miR-302e	FcεRI PMA/IONO	Decrease cytokine secretion	RelA	96
miR-146	FcεRI	Reduces activation	TRAF6, IRAK1	97– 99
miR-143	Allergic inflammation	Down-regulate allergic response	IL-13Rα1	100
miR-20a	PMA/IONO	Activate mast cells (MCs) Inhibit production of pro-inflammatory cytokines	HDAC4	101
miR-4443	T cell–derived microvesicle	Increase ERK phosphorylation and IL-8 release	PTPRJ	102– 108
miR-490	HCV-E2	Inhibits tumor metastasis		109
miR-9		Increase invasion of neoplastic MCs		110
miR-122	Tumor response	Decrease activation	SOCS1	111

Shefler *et al*. showed that MCs are activated by interaction with activated T cells or their microvesicles (mvT*s) ^105, 106^. The physical contact of MCs with activated T cells or with mvT*s induces Ras activation and ERK phosphorylation, leading to degranulation and release of several cytokines in MCs ^106– 110^. The same group later found that miR-4443 in mvT*s targets the expression of protein tyrosine phosphatase receptor type J (PTPRJ), a known ERK inhibitor ^111^. Several miRNAs that play a role in cancer have recently been discovered: miR-9 increases the invasion of neoplastic MCs ^112^. miR-122 targets SOCS1 mRNA and regulates cellular interactions involving cancer cells, MCs, and macrophages during allergic inflammation ^113^. Exosomal miRNAs have emerged as mediators of the interaction between MCs and tumor cells. MCs can inhibit hepatocellular carcinoma cell metastasis by inhibiting the ERK1/2 pathway by transferring the exosomal shuttle microRNAs, including miR-490, into hepatocellular carcinoma cells ^114^.

## Perspectives on mast cells in diseases

Traditionally, MCs have been implicated in allergic diseases. Efficacy of omalizumab—humanized anti-IgE monoclonal antibody (mAb)—and mAbs targeting Th2 cytokines or Th2 cytokine receptors for the treatment of asthma and other allergic diseases supports crucial pathogenic roles for MCs in these diseases ^115– 117^. Among the mAbs targeting cytokine/receptors, the most illustrative example is dupilumab (mAb for IL4Rα, the subunit shared by IL-4 and IL-13 receptors). This mAb blocks the functions of both IL-4 and IL-13 and is highly efficacious for the treatment of atopic dermatitis ^118^ and asthma ^119, 120^. However, effects of dupilumab likely reflect pleiotropic functions of IL-4 and IL-13 in immune and non-immune cells.

MCs are considered an important player in inflammation-associated diseases in general, as recent studies have extended their potential role in other diseases. For example, MCs seem to be involved in gastrointestinal diseases such as inflammatory bowel disease, celiac disease, and irritable bowel syndrome ^121^. The phenotype and the activation status of MCs rather than the absolute numbers in the intestinal mucosa are important for the development and progression of the diseases ^122^. MCs might also play a role in atherosclerosis. Immunohistochemical studies in autopsied human subjects and studies in murine atherosclerotic models have collectively provided evidence that the compounds released by activated MCs might promote atherogenesis at various stages during the development of atherosclerotic lesions ^123^. MCs can be pro-tumorigenic and anti-tumorigenic ^4, 124^. A recent study found that immune cells such as MCs, tumor-associated neutrophils, tumor-infiltrating macrophages, and myeloid-derived suppressor cells promote prostate cancer via various types of intercellular signaling ^125^. With regard to neural diseases, MCs might contribute to modulate the intensity of the associated depressive and anxiogenic component on the neuronal and microglial biological front ^126^. Preclinical evidence suggests that the intestinal microbiota contributes significantly to behavioral and mood disorders. Microbiotic conditions have been linked to pain, anxiety, stress, and depression in humans ^126^. Far from being substantiated by other studies, symptoms of autism spectrum disorder might also be caused by the mediators derived from MCs which could activate microglia, causing localized inflammation ^127^. MCs might play a significant role as a neuroimmune connection between these components. The next decade might see unexpected developments in MC research and their clinical translations.

## References

[1] Pejler G Abrink M Ringvall M: Mast cell proteases. *Adv Immunol.* 2007;95:167–255. 10.1016/S0065-2776(07)95006-3 17869614

[2] Dwyer DF Barrett NA Austen KF: Expression profiling of constitutive mast cells reveals a unique identity within the immune system. *Nat Immunol.* 2016;17(7):878–87. 10.1038/ni.3445 27135604 PMC5045264

[3] Klein O Sagi-Eisenberg R: Anaphylactic Degranulation of Mast Cells: Focus on Compound Exocytosis. *J Immunol Res.* 2019;2019:9542656. 10.1155/2019/9542656 31011586 PMC6442490

[4] Varricchi G Galdiero MR Loffredo S: Are Mast Cells MASTers in Cancer? *Front Immunol.* 2017;8:424. 10.3389/fimmu.2017.00424 28446910 PMC5388770

[5] Igawa S Di Nardo A: Skin microbiome and mast cells. *Transl Res.* 2017;184:68–76. 10.1016/j.trsl.2017.03.003 28390799 PMC5538027

[6] Zuani M Secco C Frossi B: Mast cells at the crossroads of microbiota and IBD. *Eur J Immunol.* 2018;48(12):1929–37. 10.1002/eji.201847504 30411335

[7] Yu W Freeland DMH Nadeau KC: Food allergy: immune mechanisms, diagnosis and immunotherapy. *Nat Rev Immunol.* 2016;16(12):751–65. 10.1038/nri.2016.111 27795547 PMC5123910

[8] Tordesillas L Berin MC Sampson HA: Immunology of Food Allergy. *Immunity.* 2017;47(1):32–50. 10.1016/j.immuni.2017.07.004 28723552

[9] Kinet JP: The high-affinity IgE receptor (Fc epsilon RI): from physiology to pathology. *Annu Rev Immunol.* 1999;17:931–72. 10.1146/annurev.immunol.17.1.931 10358778

[10] Gould HJ Sutton BJ Beavil AJ: The biology of IGE and the basis of allergic disease. *Annu Rev Immunol.* 2003;21:579–628. 10.1146/annurev.immunol.21.120601.141103 12500981

[11] Gilfillan AM Tkaczyk C: Integrated signalling pathways for mast-cell activation. *Nat Rev Immunol.* 2006;6(3):218–30. 10.1038/nri1782 16470226

[12] Gilfillan AM Rivera J: The tyrosine kinase network regulating mast cell activation. *Immunol Rev.* 2009;228(1):149–69. 10.1111/j.1600-065X.2008.00742.x 19290926 PMC2669301

[13] Jensen-Jarolim E Mechtcheriakova D Pali-Scholl I: Cancer and IgE. Introducing the Concept of AllergoOncology. (eds E Jensen-Jarolim & Penichet ML) Springer,2010;231–254. 10.1007/978-1-60761-451-7

[14] Pali-Schöll I Jensen-Jarolim E: The concept of allergen-associated molecular patterns (AAMP). *Curr Opin Immunol.* 2016;42:113–8. 10.1016/j.coi.2016.08.004 27619413

[15] Yang Z Sullivan BM Allen CD: Fluorescent *in vivo* detection reveals that IgE(+) B cells are restrained by an intrinsic cell fate predisposition. *Immunity.* 2012;36(5)857–72. 10.1016/j.immuni.2012.02.009 22406270

[16] He JS Meyer-Hermann M Xiangying D: The distinctive germinal center phase of IgE+ B lymphocytes limits their contribution to the classical memory response. *J Exp Med.* 2013;210(12):2755–71. 10.1084/jem.20131539 24218137 PMC3832920

[17] Laffleur B Duchez S Tarte K: Self-Restrained B Cells Arise following Membrane IgE Expression. *Cell Rep.* 2015;10(6):900–9. 10.1016/j.celrep.2015.01.023 25683713

[18] He JS Narayanan S Subramaniam S: Biology of IgE production: IgE cell differentiation and the memory of IgE responses. *Curr Top Microbiol Immunol.* 2015;388:1–19. 10.1007/978-3-319-13725-4_1 25553792

[19] Xiong H Dolpady J Wabl M: Sequential class switching is required for the generation of high affinity IgE antibodies. *J Exp Med.* 2012;209(2):353–64. 10.1084/jem.20111941 22249450 PMC3280879

[20] Haniuda K Fukao S Kodama T: Autonomous membrane IgE signaling prevents IgE-memory formation. *Nat Immunol.* 2016;17(9):1109–17. 10.1038/ni.3508 27428827

[21] Finkelman FD Katona IM Urban JF Jr: IL-4 is required to generate and sustain *in vivo* IgE responses. *J Immunol.* 1988;141(7):2335–41. 2459206

[22] Crotty S: T follicular helper cell differentiation, function, and roles in disease. *Immunity.* 2014;41(4):529–42. 10.1016/j.immuni.2014.10.004 25367570 PMC4223692

[23] Kobayashi T Iijima K Dent AL: Follicular helper T cells mediate IgE antibody response to airborne allergens. *J Allergy Clin Immunol.* 2017;139(1):300–313.e7. 10.1016/j.jaci.2016.04.021 27325434 PMC5115999

[24] Gowthaman U Chen JS Zhang B: Identification of a T follicular helper cell subset that drives anaphylactic IgE. *Science.* 2019;365(6456): pii: eaaw6433. 10.1126/science.aaw6433 31371561 PMC6901029

[25] Hsu C MacGlashan D Jr: IgE antibody up-regulates high affinity IgE binding on murine bone marrow-derived mast cells. *Immunol Lett.* 1996;52(2–3):129–34. 10.1016/0165-2478(96)02599-0 8905407

[26] Yamaguchi M Lantz CS Oettgen HC: IgE enhances mouse mast cell Fc(epsilon)RI expression *in vitro* and *in vivo*: evidence for a novel amplification mechanism in IgE-dependent reactions. *J Exp Med.* 1997;185(4):663–72. 10.1084/jem.185.4.663 9034145 PMC2196143

[27] Asai K Kitaura J Kawakami Y: Regulation of mast cell survival by IgE. *Immunity.* 2001;14(6):791–800. 10.1016/s1074-7613(01)00157-1 11420048

[28] Kalesnikoff J Huber M Lam V: Monomeric IgE stimulates signaling pathways in mast cells that lead to cytokine production and cell survival. *Immunity.* 2001;14(6):801–11. 10.1016/s1074-7613(01)00159-5 11420049

[29] Zellweger F Buschor P Hobi G: IL-3 but not monomeric IgE regulates FcεRI levels and cell survival in primary human basophils. *Cell Death Dis.* 2018;9(5):510. 10.1038/s41419-018-0526-9 29724998 PMC5938712

[30] Schmitz J Owyang A Oldham E: IL-33, an interleukin-1-like cytokine that signals via the IL-1 receptor-related protein ST2 and induces T helper type 2-associated cytokines. *Immunity.* 2005;23(5):479–90. 10.1016/j.immuni.2005.09.015 16286016

[31] Hammad H Chieppa M Perros F: House dust mite allergen induces asthma via Toll-like receptor 4 triggering of airway structural cells. *Nat Med.* 2009;15(4):410–6. 10.1038/nm.1946 19330007 PMC2789255

[32] Moussion C Ortega N Girard JP: The IL-1-like cytokine IL-33 is constitutively expressed in the nucleus of endothelial cells and epithelial cells *in vivo*: a novel 'alarmin'? *PLoS One.* 2008;3(10):e3331. 10.1371/journal.pone.0003331 18836528 PMC2556082

[33] Wood IS Wang B Trayhurn P: IL-33, a recently identified interleukin-1 gene family member, is expressed in human adipocytes. *Biochem Biophys Res Commun.* 2009;384(1):105–9. 10.1016/j.bbrc.2009.04.081 19393621

[34] Lefrançais E Cayrol C: Mechanisms of IL-33 processing and secretion: differences and similarities between IL-1 family members. *Eur Cytokine Netw.* 2012;23(4):120–7. 10.1684/ecn.2012.0320 23306193

[35] Roy A Ganesh G Sippola H: Mast cell chymase degrades the alarmins heat shock protein 70, biglycan, HMGB1, and interleukin-33 (IL-33) and limits danger-induced inflammation. *J Biol Chem.* 2014;289(1):237–50. 10.1074/jbc.M112.435156 24257755 PMC3879547

[36] Pinto SM Subbannayya Y Rex DA: A network map of IL-33 signaling pathway. *J Cell Commun Signal.* 2018;12(3):615–24. 10.1007/s12079-018-0464-4 29705949 PMC6039344

[37] Kakkar R Lee RT: The IL-33/ST2 pathway: therapeutic target and novel biomarker. *Nat Rev Drug Discov.* 2008;7(10):827–40. 10.1038/nrd2660 18827826 PMC4277436

[38] Komai-Koma M Brombacher F Pushparaj PN: Interleukin-33 amplifies IgE synthesis and triggers mast cell degranulation via interleukin-4 in naïve mice. *Allergy.* 2012;67(9):1118–26. 10.1111/j.1398-9995.2012.02859.x 22702477 PMC3660789

[39] Allakhverdi Z Smith DE Comeau MR: Cutting edge: The ST2 ligand IL-33 potently activates and drives maturation of human mast cells. *J Immunol.* 2007;179(4):2051–4. 10.4049/jimmunol.179.4.2051 17675461

[40] Guo Z Wu J Zhao J: IL-33 promotes airway remodeling and is a marker of asthma disease severity. *J Asthma.* 2014;51(8):863–9. 10.3109/02770903.2014.921196 24796648

[41] Reh DD Wang Y Ramanathan M Jr: Treatment-recalcitrant chronic rhinosinusitis with polyps is associated with altered epithelial cell expression of interleukin-33. *Am J Rhinol Allergy.* 2010;24(2):105–9. 10.2500/ajra.2010.24.3446 20338108 PMC2904061

[42] Liew FY Girard JP Turnquist HR: Interleukin-33 in health and disease. *Nat Rev Immunol.* 2016;16(11):676–89. 10.1038/nri.2016.95 27640624

[43] Saluja R Khan M Church MK: The role of IL-33 and mast cells in allergy and inflammation. *Clin Transl Allergy.* 2015;5:33. 10.1186/s13601-015-0076-5 26425339 PMC4588911

[44] Makrinioti H Toussaint M Jackson DJ: Role of interleukin 33 in respiratory allergy and asthma. *Lancet Respir Med.* 2014;2(3):226–37. 10.1016/S2213-2600(13)70261-3 24621684

[45] Sjöberg LC Gregory JA Dahlén SE: Interleukin-33 exacerbates allergic bronchoconstriction in the mice via activation of mast cells. *Allergy.* 2015;70(5):514–21. 10.1111/all.12590 25660244

[46] Teufelberger AR Nordengrün M Braun H: The IL-33/ST2 axis is crucial in type 2 airway responses induced by *Staphylococcus aureus*-derived serine protease-like protein D. *J Allergy Clin Immunol.* 2018;141(2):549–559.e7. 10.1016/j.jaci.2017.05.004 28532656

[47] Liu T Kanaoka Y Barrett NA: Aspirin-Exacerbated Respiratory Disease Involves a Cysteinyl Leukotriene-Driven IL-33-Mediated Mast Cell Activation Pathway. *J Immunol.* 2015;195(8):3537–45. 10.4049/jimmunol.1500905 26342029 PMC4592820

[48] Drube S Kraft F Dudeck J: MK2/3 Are Pivotal for IL-33-Induced and Mast Cell-Dependent Leukocyte Recruitment and the Resulting Skin Inflammation. *J Immunol.* 2016;197(9):3662–8. 10.4049/jimmunol.1600658 27694493

[49] Noval Rivas M Burton OT Oettgen HC: IL-4 production by group 2 innate lymphoid cells promotes food allergy by blocking regulatory T-cell function. *J Allergy Clin Immunol.* 2016;138(3):801–811.e9. 10.1016/j.jaci.2016.02.030 27177780 PMC5014699

[50] Leyva-Castillo JM Galand C Kam C: Mechanical Skin Injury Promotes Food Anaphylaxis by Driving Intestinal Mast Cell Expansion. *Immunity.* 2019;50(5):1262–1275.e4. 10.1016/j.immuni.2019.03.023 31027995 PMC6531322

[51] Galand C Leyva-Castillo JM Yoon J: IL-33 promotes food anaphylaxis in epicutaneously sensitized mice by targeting mast cells. *J Allergy Clin Immunol.* 2016;138(5):1356–66. 10.1016/j.jaci.2016.03.056 27372570 PMC5099088

[52] Shimokawa C Kanaya T Hachisuka M: Mast Cells Are Crucial for Induction of Group 2 Innate Lymphoid Cells and Clearance of Helminth Infections. *Immunity.* 2017;46(5):863–874.e4. 10.1016/j.immuni.2017.04.017 28514691

[53] Morita H Arae K Unno H: An Interleukin-33-Mast Cell-Interleukin-2 Axis Suppresses Papain-Induced Allergic Inflammation by Promoting Regulatory T Cell Numbers. *Immunity.* 2015;43(1):175–86. 10.1016/j.immuni.2015.06.021 26200013 PMC4533925

[54] Kawakami T Kasakura K: Mast Cell Eavesdropping on Bacterial Communications. *Cell Host Microbe.* 2019;26(1):3–5. 10.1016/j.chom.2019.06.006 31295422

[55] McNeil BD Pundir P Meeker S: Identification of a mast-cell-specific receptor crucial for pseudo-allergic drug reactions. *Nature.* 2015;519(7542):237–41. 10.1038/nature14022 25517090 PMC4359082

[56] Tatemoto K Nozaki Y Tsuda R: Endogenous protein and enzyme fragments induce immunoglobulin E-independent activation of mast cells via a G protein-coupled receptor, MRGPRX2. *Scand J Immunol.* 2018;87(5):e12655. 10.1111/sji.12655 29484687

[57] Pundir P Liu R Vasavda C: A Connective Tissue Mast-Cell-Specific Receptor Detects Bacterial Quorum-Sensing Molecules and Mediates Antibacterial Immunity. *Cell Host Microbe.* 2019;26(1):114–122.e8. 10.1016/j.chom.2019.06.003 31278040 PMC6649664

[58] Gaudenzio N Sibilano R Marichal T: Different activation signals induce distinct mast cell degranulation strategies. *J Clin Invest.* 2016;126(10):3981–98. 10.1172/JCI85538 27643442 PMC5096814

[59] Subramanian H Gupta K Guo Q: Mas-related gene X2 (MrgX2) is a novel G protein-coupled receptor for the antimicrobial peptide LL-37 in human mast cells: resistance to receptor phosphorylation, desensitization, and internalization. *J Biol Chem.* 2011;286(52):44739–49. 10.1074/jbc.M111.277152 22069323 PMC3247983

[60] Yu Y Zhang Y Zhang Y: LL-37-induced human mast cell activation through G protein-coupled receptor MrgX2. *Int Immunopharmacol.* 2017;49:6–12. 10.1016/j.intimp.2017.05.016 28549244

[61] Reinholz M Ruzicka T Schauber J: Cathelicidin LL-37: an antimicrobial peptide with a role in inflammatory skin disease. *Ann Dermatol.* 2012;24(2):126–35. 10.5021/ad.2012.24.2.126 22577261 PMC3346901

[62] Muto Y Wang Z Vanderberghe M: Mast cells are key mediators of cathelicidin-initiated skin inflammation in rosacea. *J Invest Dermatol.* 2014;134(11):2728–36. 10.1038/jid.2014.222 24844861 PMC4199909

[63] Manorak W Idahosa C Gupta K: Upregulation of Mas-related G Protein coupled receptor X2 in asthmatic lung mast cells and its activation by the novel neuropeptide hemokinin-1. *Respir Res.* 2018;19(1):1. 10.1186/s12931-017-0698-3 29295703 PMC5751818

[64] Fujisawa D Kashiwakura J Kita H: Expression of Mas-related gene X2 on mast cells is upregulated in the skin of patients with severe chronic urticaria. *J Allergy Clin Immunol.* 2014;134(3):622–633.e9. 10.1016/j.jaci.2014.05.004 24954276

[65] Meixiong J Anderson M Limjunyawong N: Activation of Mast-Cell-Expressed Mas-Related G-Protein-Coupled Receptors Drives Non-histaminergic Itch. *Immunity.* 2019;50(5):1163–1171.e5. 10.1016/j.immuni.2019.03.013 31027996 PMC6531358

[66] Vocanson M Hennino A Rozières A: Effector and regulatory mechanisms in allergic contact dermatitis. *Allergy.* 2009;64(12):1699–714. 10.1111/j.1398-9995.2009.02082.x 19839974

[67] Arizono N Matsuda S Hattori T: Anatomical variation in mast cell nerve associations in the rat small intestine, heart, lung, and skin. Similarities of distances between neural processes and mast cells, eosinophils, or plasma cells in the jejunal lamina propria. *Lab Invest.* 1990;62(5):626–34. 2342332

[68] Bienenstock J MacQueen G Sestini P: Mast cell/nerve interactions *in vitro* and *in vivo*. *Am Rev Respir Dis.* 1991;143(3 Pt 2):S55–8. 10.1164/ajrccm/143.3_Pt_2.S55 2003692

[69] Purcell WM Atterwill CK: Mast cells in neuroimmune function: neurotoxicological and neuropharmacological perspectives. *Neurochem Res.* 1995;20(5):521–32. 10.1007/bf01694534 7643958

[70] Kawakami Y Yumoto K Kawakami T: An improved mouse model of atopic dermatitis and suppression of skin lesions by an inhibitor of Tec family kinases. *Allergol Int.* 2007;56(4):403–9. 10.2332/allergolint.O-07-486 17713360

[71] Serhan N Basso L Sibilano R: House dust mites activate nociceptor-mast cell clusters to drive type 2 skin inflammation. *Nat Immunol.* 2019;20(11):1435–43. 10.1038/s41590-019-0493-z 31591569 PMC6858877

[72] Solinski HJ Kriegbaum MC Tseng PY: Nppb Neurons Are Sensors of Mast Cell-Induced Itch. *Cell Rep.* 2019;26(13):3561–3573.e4. 10.1016/j.celrep.2019.02.089 30917312 PMC6490177

[73] Green DP Limjunyawong N Gour N: A Mast-Cell-Specific Receptor Mediates Neurogenic Inflammation and Pain. *Neuron.* 2019;101(13):412–420.e3. 10.1016/j.neuron.2019.01.012 30686732 PMC6462816

[74] Roy S Ganguly A Haque M: Angiogenic Host Defense Peptide AG-30/5C and Bradykinin B _2_ Receptor Antagonist Icatibant Are G Protein Biased Agonists for MRGPRX2 in Mast Cells. *J Immunol.* 2019;202(4):1229–38. 10.4049/jimmunol.1801227 30651343 PMC6369923

[75] Babina M Guhl S Artuc M: Allergic FcεRI- and pseudo-allergic MRGPRX2-triggered mast cell activation routes are independent and inversely regulated by SCF. *Allergy.* 2018;73(1):256–60. 10.1111/all.13301 28859248

[76] Babina M Wang Z Artuc M: MRGPRX2 is negatively targeted by SCF and IL-4 to diminish pseudo-allergic stimulation of skin mast cells in culture. *Exp Dermatol.* 2018;27(11):1298–303. 10.1111/exd.13762 30091263

[77] Cop N Ebo DG Bridts CH: Influence of IL-6, IL-33, and TNF-α on human mast cell activation: Lessons from single cell analysis by flow cytometry. *Cytometry B Clin Cytom.* 2018;94(3):405–11. 10.1002/cyto.b.21547 28802100

[78] Babina M Wang Z Franke K: Yin-Yang of IL-33 in Human Skin Mast Cells: Reduced Degranulation, but Augmented Histamine Synthesis through p38 Activation. *J Invest Dermatol.* 2019;139(7):1516–1525.e3. 10.1016/j.jid.2019.01.013 30684550

[79] Wang Z Guhl S Franke K: IL-33 and MRGPRX2-Triggered Activation of Human Skin Mast Cells-Elimination of Receptor Expression on Chronic Exposure, but Reinforced Degranulation on Acute Priming. *Cells.* 2019;8(4): pii: E341. 10.3390/cells8040341 30979016 PMC6523246

[80] Widiapradja A Manteufel EJ Dehlin HM: Regulation of Cardiac Mast Cell Maturation and Function by the Neurokinin-1 Receptor in the Fibrotic Heart. *Sci Rep.* 2019;9(1):11004. 10.1038/s41598-019-47369-0 31358823 PMC6662794

[81] Bartel DP: MicroRNAs: target recognition and regulatory functions. *Cell.* 2009;136(2):215–33. 10.1016/j.cell.2009.01.002 19167326 PMC3794896

[82] Chiang HR Schoenfeld LW Ruby JG: Mammalian microRNAs: experimental evaluation of novel and previously annotated genes. *Genes Dev.* 2010;24(10):992–1009. 10.1101/gad.1884710 20413612 PMC2867214

[83] Shefler I Salamon P Mekori YA: MicroRNA Involvement in Allergic and Non-Allergic Mast Cell Activation. *Int J Mol Sci.* 2019;20(9):2145. 10.3390/ijms20092145 31052286 PMC6539777

[84] Yamada Y Kosaka K Miyazawa T: miR-142-3p enhances FcεRI-mediated degranulation in mast cells. *Biochem Biophys Res Commun.* 2014;443(3):980–6. 10.1016/j.bbrc.2013.12.078 24361879

[85] Monticelli S Ansel KM Xiao C: MicroRNA profiling of the murine hematopoietic system. *Genome Biol.* 2005;6(8):R71. 10.1186/gb-2005-6-8-r71 16086853 PMC1273638

[86] Teng Y Zhang R Yu H: Altered MicroRNA Expression Profiles in Activated Mast Cells Following IgE-FcεRI Cross-Linking with Antigen. *Cell Physiol Biochem.* 2015;35(6):2098–110. 10.1159/000374016 25895812

[87] Biethahn K Orinska Z Vigorito E: miRNA-155 controls mast cell activation by regulating the PI3Kγ pathway and anaphylaxis in a mouse model. *Allergy.* 2014;69(6):752–62. 10.1111/all.12407 24734904

[88] Abebayehu D Spence AJ Qayum AA: Lactic Acid Suppresses IL-33-Mediated Mast Cell Inflammatory Responses via Hypoxia-Inducible Factor-1α-Dependent miR-155 Suppression. *J Immunol.* 2016;197(7):2909–17. 10.4049/jimmunol.1600651 27559047 PMC5026940

[89] Wang Z Yi T Long M: Involvement of the Negative Feedback of IL-33 Signaling in the Anti-Inflammatory Effect of Electro-acupuncture on Allergic Contact Dermatitis via Targeting MicroRNA-155 in Mast Cells. *Inflammation.* 2018;41(3):859–69. 10.1007/s10753-018-0740-8 29404871

[90] Qayum AA Paranjape A Abebayehu D: IL-10-Induced miR-155 Targets SOCS1 To Enhance IgE-Mediated Mast Cell Function. *J Immunol.* 2016;196(11):4457–67. 10.4049/jimmunol.1502240 27183599 PMC4875869

[91] Li Y Liu J Zhang J: Characterization of microRNA profile in IgE-mediated mouse BMMCs degranulation. *J Microbiol Immunol Infect.* 2018; pii: S1684-1182(18)30477-8. 10.1016/j.jmii.2018.10.006 30473142

[92] Bao Y Wang S Gao Y: MicroRNA-126 accelerates IgE-mediated mast cell degranulation associated with the PI3K/Akt signaling pathway by promoting Ca ^2+^ influx. *Exp Ther Med.* 2018;16(3):2763–2769. 10.3892/etm.2018.6510 30186504 PMC6122504

[93] Yang Q Xu H Yang J: MicroRNA-223 affects IL-6 secretion in mast cells via the IGF1R/PI3K signaling pathway. *Int J Mol Med.* 2016;38(2):507–12. 10.3892/ijmm.2016.2649 27354148

[94] Just J Munk Ipsen P Kruhøffer M: Human Mast Cell Sensitization with IgE Increases miRNA-210 Expression. *Int Arch Allergy Immunol.* 2019;179(2):102–7. 10.1159/000496513 30965334

[95] Li W Liu F Wang J: MicroRNA-21-Mediated Inhibition of Mast Cell Degranulation Involved in the Protective Effect of Berberine on 2,4-Dinitrofluorobenzene-Induced Allergic Contact Dermatitis in Rats via p38 Pathway. *Inflammation.* 2018;41(2):689–99. 10.1007/s10753-017-0723-1 29282578

[96] Mayoral RJ Deho L Rusca N: MiR-221 influences effector functions and actin cytoskeleton in mast cells. *PLoS One.* 2011;6(10):e26133. 10.1371/journal.pone.0026133 22022537 PMC3192147

[97] Mayoral RJ Pipkin ME Pachkov M: MicroRNA-221-222 regulate the cell cycle in mast cells. *J Immunol.* 2008;182(1):433–45. 10.4049/jimmunol.182.1.433 19109175 PMC2610349

[98] Zhou Y Yang Q Xu H: miRNA-221-3p Enhances the Secretion of Interleukin-4 in Mast Cells through the Phosphatase and Tensin Homolog/p38/Nuclear Factor-kappaB Pathway. *PLoS One.* 2016;11(2):e0148821. 10.1371/journal.pone.0148821 26901347 PMC4764704

[99] Xiao L Jiang L Hu Q: MiR-302e attenuates allergic inflammation *in vitro* model by targeting RelA. *Biosci Rep.* 2018;38(3): pii: BSR20180025. 10.1042/BSR20180025 29748238 PMC6435536

[100] Rusca N Dehò L Montagner S: MiR-146a and NF-κB1 regulate mast cell survival and T lymphocyte differentiation. *Mol Cell Biol.* 2012;32(21):4432–44. 10.1128/MCB.00824-12 22927641 PMC3486148

[101] Taganov KD Boldin MP Chang KJ: NF-kappaB-dependent induction of microRNA miR-146, an inhibitor targeted to signaling proteins of innate immune responses. *Proc Natl Acad Sci U S A.* 2006;103(33):12481–6. 10.1073/pnas.0605298103 16885212 PMC1567904

[102] Yang L Boldin MP Yu Y: miR-146a controls the resolution of T cell responses in mice. *J Exp Med.* 2012;209(9):1655–70. 10.1084/jem.20112218 22891274 PMC3428948

[103] Yu S Zhang R Zhu C: MicroRNA-143 downregulates interleukin-13 receptor alpha1 in human mast cells. *Int J Mol Sci.* 2013;14(8):16958–69. 10.3390/ijms140816958 23965966 PMC3759945

[104] Lu Y Li Z Xie B: hsa-miR-20a-5p attenuates allergic inflammation in HMC-1 cells by targeting HDAC4. *Mol Immunol.* 2019;107:84–90. 10.1016/j.molimm.2019.01.010 30684894

[105] Shefler I Pasmanik-Chor M Kidron D: T cell-derived microvesicles induce mast cell production of IL-24: relevance to inflammatory skin diseases. *J Allergy Clin Immunol.* 2014;133(1):217–224.e3–3. 10.1016/j.jaci.2013.04.035 23768573

[106] Shefler I Salamon P Reshef T: T cell-induced mast cell activation: a role for microparticles released from activated T cells. *J Immunol.* 2010;185(7):4206–12. 10.4049/jimmunol.1000409 20810987

[107] Baram D Vaday GG Salamon P: Human mast cells release metalloproteinase-9 on contact with activated T cells: juxtacrine regulation by TNF-alpha. *J Immunol.* 2001;167(7):4008–16. 10.4049/jimmunol.167.7.4008 11564820

[108] Mor A Shefler I Salamon P: Characterization of ERK activation in human mast cells stimulated by contact with T cells. *Inflammation.* 2010;33(2):119–25. 10.1007/s10753-009-9165-8 19908133

[109] Salamon P Shoham NG Puxeddu I: Human mast cells release oncostatin M on contact with activated T cells: possible biologic relevance. *J Allergy Clin Immunol.* 2008;121(2):448–455.e5. 10.1016/j.jaci.2007.08.054 18028996

[110] Shefler I Mekori YA Mor A: Stimulation of human mast cells by activated T cells leads to N-Ras activation through Ras guanine nucleotide releasing protein 1. *J Allergy Clin Immunol.* 2008;122(6):1222–5. 10.1016/j.jaci.2008.07.024 18760455

[111] Shefler I Salamon P Levi-Schaffer F: MicroRNA-4443 regulates mast cell activation by T cell-derived microvesicles. *J Allergy Clin Immunol.* 2018;141(6):2132–2141.e4. 10.1016/j.jaci.2017.06.045 28823811

[112] Fenger JM Bear MD Volinia S: Overexpression of miR-9 in mast cells is associated with invasive behavior and spontaneous metastasis. *BMC Cancer.* 2014;14:84. 10.1186/1471-2407-14-84 24517413 PMC3933481

[113] Noh K Kim M Kim Y: miR-122-SOCS1-JAK2 axis regulates allergic inflammation and allergic inflammation-promoted cellular interactions. *Oncotarget.* 2017;8(38):63155–63176. 10.18632/oncotarget.19149 28968979 PMC5609911

[114] Xiong L Zhen S Yu Q: HCV-E2 inhibits hepatocellular carcinoma metastasis by stimulating mast cells to secrete exosomal shuttle microRNAs. *Oncol Lett.* 2017;14(2):2141–2146. 10.3892/ol.2017.6433 28781655 PMC5530191

[115] Kawakami T Blank U: From IgE to Omalizumab. *J Immunol.* 2016;197(11):4187–4192. 10.4049/jimmunol.1601476 27864548 PMC5123831

[116] Holgate ST Chuchalin AG Hébert J: Efficacy and safety of a recombinant anti-immunoglobulin E antibody (omalizumab) in severe allergic asthma. *Clin Exp Allergy.* 2004;34(4):632–8. 10.1111/j.1365-2222.2004.1916.x 15080818

[117] Maurer M Rosén K Hsieh HJ: Omalizumab for the treatment of chronic idiopathic or spontaneous urticaria. *N Engl J Med.* 2013;368(10):924–35. 10.1056/NEJMoa1215372 23432142

[118] Simpson EL Bieber T Guttman-Yassky E: Two Phase 3 Trials of Dupilumab versus Placebo in Atopic Dermatitis. *N Engl J Med.* 2016;375(24):2335–48. 10.1056/NEJMoa1610020 27690741

[119] Castro M Corren J Pavord ID: Dupilumab Efficacy and Safety in Moderate-to-Severe Uncontrolled Asthma. *N Engl J Med.* 2018;378(26):2486–96. 10.1056/NEJMoa1804092 29782217

[120] Rabe KF Nair P Brusselle G: Efficacy and Safety of Dupilumab in Glucocorticoid-Dependent Severe Asthma. *N Engl J Med.* 2018;378(26):2475–85. 10.1056/NEJMoa1804093 29782224

[121] Frossi B De Carli M Calabrò A: Coeliac Disease and Mast Cells. *Int J Mol Sci.* 2019;20(14): pii: E3400. 10.3390/ijms20143400 31373285 PMC6678566

[122] Theoharides TC Valent P Akin C: Mast Cells, Mastocytosis, and Related Disorders. *N Engl J Med.* 2015;373(19):1885–6. 10.1056/NEJMc1510021 26535528

[123] Kovanen PT: Mast Cells as Potential Accelerators of Human Atherosclerosis-From Early to Late Lesions. *Int J Mol Sci.* 2019;20(18): pii: E4479. 10.3390/ijms20184479 31514285 PMC6770933

[124] Derakhshani A Vahidian F Alihasanzadeh M: Mast cells: A double-edged sword in cancer. *Immunol Lett.* 2019;209:28–35. 10.1016/j.imlet.2019.03.011 30905824

[125] Hayashi T Fujita K Matsushita M: Main Inflammatory Cells and Potentials of Anti-Inflammatory Agents in Prostate Cancer. *Cancers (Basel).* 2019;11(8): pii: E1153. 10.3390/cancers11081153 31408948 PMC6721573

[126] Traina G: Mast Cells in Gut and Brain and Their Potential Role as an Emerging Therapeutic Target for Neural Diseases. *Front Cell Neurosci.* 2019;13:345. 10.3389/fncel.2019.00345 31417365 PMC6682652

[127] Theoharides TC Tsilioni I Patel AB: Atopic diseases and inflammation of the brain in the pathogenesis of autism spectrum disorders. *Transl Psychiatry.* 2016;6(6):e844. 10.1038/tp.2016.77 27351598 PMC4931610

